# A new species of *Tychobythinus* Ganglbauer, 1896 (Coleoptera: Staphylinidae: Pselaphinae) from Turkey

**DOI:** 10.3897/BDJ.1.e963

**Published:** 2013-09-16

**Authors:** Rostislav Bekchiev

**Affiliations:** † National Museum of Natural History, Sofia, Bulgaria

**Keywords:** Pselaphinae, Bythinini, *Tychobythinus oculatus*, new species, taxonomy, Turkey, Asia

## Abstract

A new species of the genus *Tychobythinus* Ganglbauer, 1896, *Tychobythinus oculatus* sp. n., is described from near Köyceğiz, Muğla Province, in southwestern Turkey. The new species is morphologically closely related to *Tychobythinus abnormipes* Reitter, 1910, and *Tychobythinus pauper* Kiesenwetter, 1858, but can be readily distinguished from both by having very simplified internal armature of the aedeagus, and by the specific shape of the gular depression.

## Introduction

The genus *Tychobythinus* includes about 84 known species from the Palaearctic region (Besuchet 2008, Hlaváč and Jalžić 2009, Löbl and Besuchet 2004). Almost all species are rarely collected and have limited distribution. Up to now only one species is known from Turkey (Besuchet 1978). A second species from Turkey was discovered in the collection of the Museum für Naturkunde der Humboldt Universität in Berlin and is described here as *Tychobythinus oculatus* sp. n. The new species differs from all known congeners mainly by the shape of gular region, and the aedeagus.

## Materials and methods

Dissections were made using standard techniques. Genitalia and small parts were mounted in Euparal or Canada balsam on acetate labels which are pinned with the specimens. All photos were taken with a Zeiss Stemi 2000 microscope equipped an AxioCam ERc 5s camera. Image stacks were processed using COMBINE ZP (Hadley 2010).

The material used for this study is deposited in the following collections:

MNHB – Museum für Naturkunde der Humboldt Universität zu Berlin, Germany (Johannes Frisch)

NMNHS – National Museum of Natural History, Sofia, Bulgaria

PCVB – Personal collection Volker Brachat, Geretsried, Germany

## Taxon treatments

### Tychobythinus oculatus

Bekchiev sp. n.

urn:lsid:zoobank.org:act:9A79187E-FC75-4CB7-B634-ABBD4B471D2A

#### Materials

- **Type status:** Holotype. **Occurrence:** recordedBy: P. Wunderie, V. Assing; individualCount: 1; sex: male; **Location:** country: Turkey; verbatimLocality: Mugla, SE Köyceğiz; verbatimElevation: 10 m; locationRemarks: flood-plain wood; verbatimLatitude: 36°56'50"N; verbatimLongitude: 28°43'56"E; **Event:** eventDate: 28.03.2002; **Record Level:** institutionCode: MNHB
- **Type status:** Paratype. **Occurrence:** recordedBy: P. Wunderie, V. Assing; individualCount: 3; sex: 2 males, 1 female; **Location:** country: Turkey; verbatimLocality: Mugla, SE Köyceğiz; verbatimElevation: 10 m; verbatimLatitude: 36°56'50"N; verbatimLongitude: 28°43'56"E; **Event:** eventDate: 28.03.2002; **Record Level:** institutionCode: MNHB
- **Type status:** Paratype. **Occurrence:** recordedBy: P. Wunderie, V. Assing; individualCount: 2; sex: males; **Location:** country: Turkey; verbatimLocality: Mugla, SE Köyceğiz; verbatimElevation: 10 m; verbatimLatitude: 36°56'50"N; verbatimLongitude: 28°43'56"E; **Event:** eventDate: 28.03.2002; **Record Level:** institutionCode: NMNHS
- **Type status:** Paratype. **Occurrence:** recordedBy: P. Wunderie, V. Assing; individualCount: 7; sex: 2 males, 5 females; **Location:** country: Turkey; verbatimLocality: Mugla, SE Köyceğiz; verbatimElevation: 10 m; verbatimLatitude: 36°56'50"N; verbatimLongitude: 28°43'56"E; **Event:** eventDate: 28.03.2002; **Record Level:** institutionCode: PCVB

#### Description

Male: Body dark brown (Fig. 1a); pubescent with short, golden semierect setae and some long, erect setae. Length 1.10-1.19 mm. Head (Fig. 1b) wider than long (0.25/0.20 mm), covered with dense, semierect setae. Frontal rostrum distinctly wider than long (0.13/0.07 mm); antennal tubercles well-developed, median depression shallow. Vertex convex, with distinct median ridge. Ventral side of the head with narrow and deep depression in gular region, the depression distinctly longer than wide, shining; anterior border of depression carinate, with two obtuse teeth; posterior border simple, with one thick and long seta. Eyes well developed, each composed of 10-12 ommatidia. Maxillary palpi long (Fig. 1b), almost as long as antennae, palpomeres II–III granular, palpomeres IV with dense, short and recumbent setae. Antennae (Fig. 1b) short – 0.43-0.44 mm; scapes longer than wide (0.09-0.1/0.04-0.05 mm), with a small tubercule in anteromesal part; pedicel globular (0.034/0.034 mm); antennomeres III slightly longer than wide (0.025/0.022); antennomeres IV – VIII about same length (0.017/0.025 mm each); antenommeres IX wider than long (0.043/0.017 mm); antenommeres X wider than long (0.067/0.017 mm); XI longer than wide (0.12/0.068 mm). Pronotum convex (0.27/0.30 mm), widest part before middle, covered with dense, long, sеmierected setation; disk shiny; lateral antebasal foveae well-defined, connected by well-defined antebasal sulcus. Elytra wider than long (0.510/0.425 mm) each with two basal foveae, sutural stria well-defined through whole length of elytron; covered with long, golden and semierect setae, with deep and irregular punctation. Abdomen slightly narrower than elytra, covered with a long, semierect setae, first two visible tergites of same length. Legs long and slender, protibia simple, metatibia with strong spur in apical inner part.

**Figure 2.**
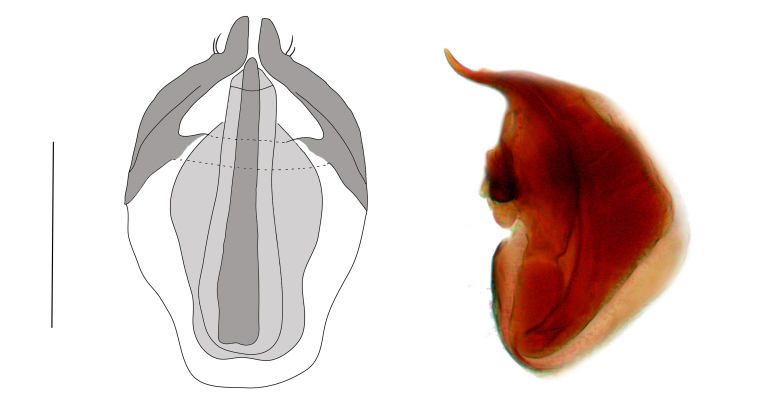
*Tychobythinus oculatus* sp.n., aedeagus - dorsal and lateral view (scale: 0.1 mm).

Aedeagus as in Fig. 2, length – 0.20-0.22 mm.

Sexual dimorphism: The female is with a simple scapes, gular region of the head is without modifications, metatibia is simple.

#### Diagnosis

*Tychobythinus oculatus* sp. n. is morphologically closely related to *Tychobythinus abnormipes* Reitter, 1910, and *Tychobythinus pauper* Kiesenwetter, 1858, both from Greece, they share similar shape of the scape (longer than wide, with a small tubercule). The new species clearly differs from *Tychobythinus abnormipes*, and *Tychobythinus pauper* by the very simplified internal armature of the aedeagus (in *Tychobythinus abnormipes* and *Tychobythinus pauper* the aedeagus has a long and crossed internal aphophyses), and by the shape of the gular depression (simplified and narrow in *Tychobythinus oculatus*; wide and triangular in *Tychobythinus abnormipes*; strongly modified in *Tychobythinus pauper*). *Tychobythinus oculatus* sp.n. can be readily distinguished from *Tychobythinus vignai*, the only other currently known species from Turkey, by the presence of eyes (related to its way of life) and by the specific shapes of the antennae, gular region and aedeagus.

#### Etymology

Ocultus means ‘having eyes’, to distinguish it from the only other hitherto known species of *Tychobythinus* from Turkey (*Tychobythinus vignai* Besuchet, 1987) which is eyeless.

#### Distribution

Turkey.

## Supplementary Material

XML Treatment for Tychobythinus oculatus

## Figures and Tables

**Figure 1a.**
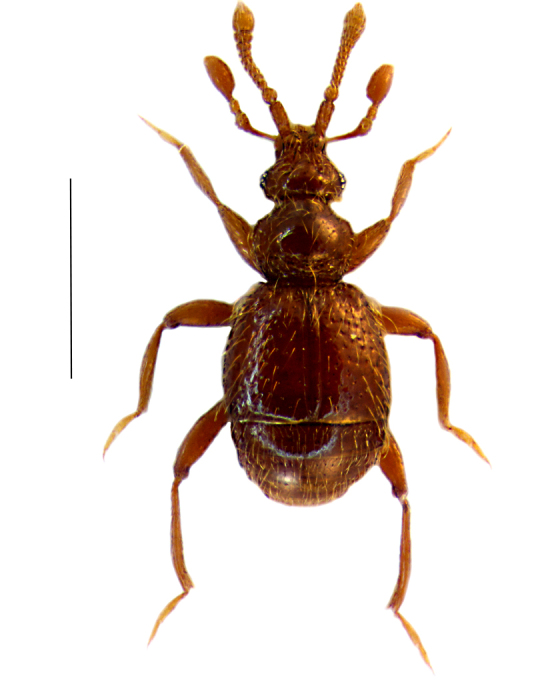
habitus, scale: 0.6 mm

**Figure 1b.**
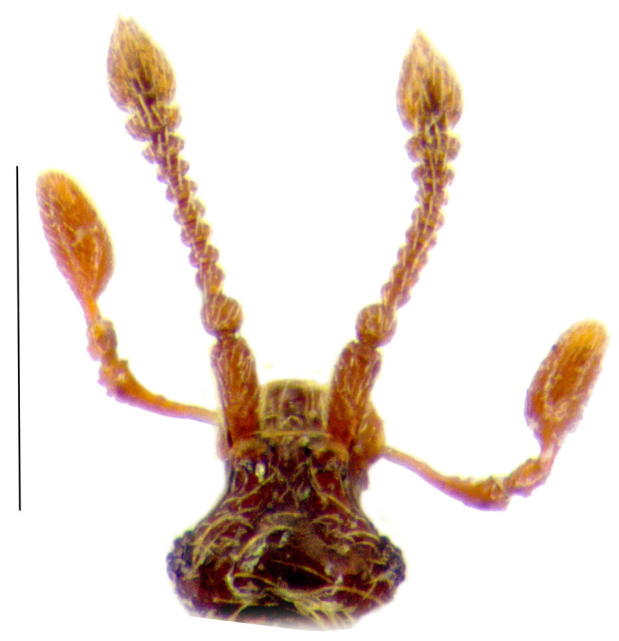
head, scale: 0.3 mm
